# Valorization of fish from the Adriatic Sea: nutritional properties and shelf life prolongation of *Aphia minuta* through essential oils

**DOI:** 10.3389/fnut.2024.1454228

**Published:** 2024-08-23

**Authors:** Rosaria Marino, Marzia Albenzio, Antonella della Malva, Angela Racioppo, Barbara Speranza, Antonio Bevilacqua

**Affiliations:** Department of Agriculture, Food, Natural Resources, and Engineering (DAFNE), University of Foggia, Foggia, Italy

**Keywords:** *Aphia minuta*, n-3 fatty acids, essential amino acids, essential oils, shelf life

## Abstract

This study aimed to exploit the nutritional and microbiological qualities of *Aphia minuta*, which are still largely unknown; they are collected from Golfo di Manfredonia (Adriatic Sea). Chemical composition, fatty acids, and amino acid profiles were evaluated during winter, spring, and summer (two samples each season). The protein content was highest in spring, while no significant differences were found for fat and ash contents across all sampling periods. Fatty acid profile analyses revealed that monounsaturated and polyunsaturated fatty acids were affected by the sampling season. Notably, the value of n-3 polyunsaturated fatty acids increased in spring and summer compared to the winter season. The highest content of essential amino acids was measured during the spring and summer seasons (*P* < 0.01), with leucine and lysine being the most dominant. Regardless of the fishing season, from a nutritional point of view, this species is an excellent source of bioactive compounds. This study also focused on the microbiological quality and shelf life of *Aphia minuta*. Initially, the bioactivity of three different essential oils (thymol, lemon, and citrus extract) was tested on *Pseudomonas fluorescens, Staphylococcus aureus*, and *Escherichia coli*. These essential oils were then combined with various packaging materials (conventional, maize starch, and polylactate) and packaging atmosphere (air, vacuum, and a modified atmosphere with reduced oxygen content). The results indicated that combining citrus extract with vacuum packaging significantly reduced the psychrotrophic viable count to undetectable levels after 7 days. This study suggests some important considerations for exploiting and expanding the market of the *Aphia minuta*.

## 1 Introduction

*Aphia minuta* (1) is a small neretic pelagic species, reaching a maximum size of 6 cm. It belongs to the Gobiidae family, one of the largest groups of fish in coastal marine waters. This species has a transparent or whitish-reddish body, compressed laterally, with chromatophores along the base of the median fin and on the head. It is found throughout the northeastern Atlantic region, from the western Baltic and Norway to Morocco and the Mediterranean and Black Seas (2). This species is highly valued in the Mediterranean region for its taste, making it a thriving business.

In Italy, the *Aphia minuta* fishery, referred to as “Rossetto,” has a long history and represents one of the most important small-scale activities; it is usually caught by small-scale fleets disseminated throughout the Ligurian Sea (3), Tyrrhenian Sea (4), Adriatic Sea (5, 6), and around the coast of Sicily and Sardinia (7, 8). Particularly, catches are about 160 tons per year, most of them in the southern Adriatic, and the fishing fleet comprises about 400 vessels (8).

It is known that fish provide significant amounts of bioavailable nutrients such as proteins, lipids, and micronutrients such as vitamins, iron, selenium, or zinc with well-recognized health benefits (9). In particular, fish are a good source of long-chain polyunsaturated fatty acids, supporting human health through various metabolic functions. In order to harness the potential of any food item to its fullest extent, its nutritional composition must be known. This is particularly important in the case of fish, which represent a large biodiversity with varieties of species and consequent differences in nutritional composition. To date, no studies are available on the nutritional properties of *Aphia minuta*; most studies on this fish concern the growth and reproduction aspects (5, 8, 10).

The high water activity, neutral pH, high content of low molecular weight molecules, and cold-adapted microbial flora make fish highly perishable. As a result, traditional fish preservation methods (salting, drying, and freezing) drastically reduce water activity. However, consumers now prefer fresh food that has been minimally processed. Many consumers perceive the use of synthetic preservatives as a potential health risk, so the use of natural preservatives is being investigated (11). Essential oils (EOs) are aromatic extracts (mainly terpenes and other aromatic compounds) of whole plants or specific parts of plants whose bioactive components confer biological properties (antioxidants, insecticides, antimicrobials, and so on). These properties depend on the plant and many other factors (agronomy, plant part, extraction process, application process). The solubility of the EO and the type of bacteria (gram-negative bacteria are more resistant than gram-positive bacteria) strongly influence the antimicrobial properties; furthermore, the food matrix can interfere with the activity of the EO (12). EOs are valued for food preservation because they are natural products; many are widely used and historically safe, and their addition to foods is part of the current trend toward clean labeling.

Several authors have demonstrated significant antimicrobial and antioxidant effects of various EOs. The efficacy of EOs depends on chemical structure, concentration, comparison of antimicrobial spectrum with target microorganisms, interactions with food matrix, and method of application (13). Regarding the food matrix, some authors have suggested that the fat level in fish may affect the efficacy of essential oils, as some essential oils have been reported to be more effective in low-fat fish (cod) than in fatty fish (salmon). Several systems have been tested for the application of EO to fish: (i) direct application to the food, such as dipping or direct application, (ii) as part of an active packaging, such as vapor phase application (14), or (iii) film packaging embedded in edible coatings (chitosan, starch, gelatin, blends, and others) with varying degrees of emulsification (nanoemulsions). The latter two allow a gradual release of the active compound and are more effective than direct application (12). Cooling combined with vacuum or modified atmosphere packaging are the most commonly used technologies in combination with EO to preserve fish. In this context, the use of essential oils/natural extracts, combined with packaging and modified atmosphere, could be a promising strategy to promote and valorize *Aphia minuta* commercialization.

Therefore, the main aims of this research were as follows:

a) Nutritional characterization (chemical composition, fatty acids, and amino acid profile) of *Aphia minuta* from the Adriatic Sea and its compositional changes throughout the fishing season;
b) Preliminary optimization of packaging, treatment with natural compounds, and storage conditions are needed to increase the shelf-life and promote the diffusion of this product in other regions.

## 2 Materials and methods

### 2.1 Study area and sample collection

**Figure 1:**
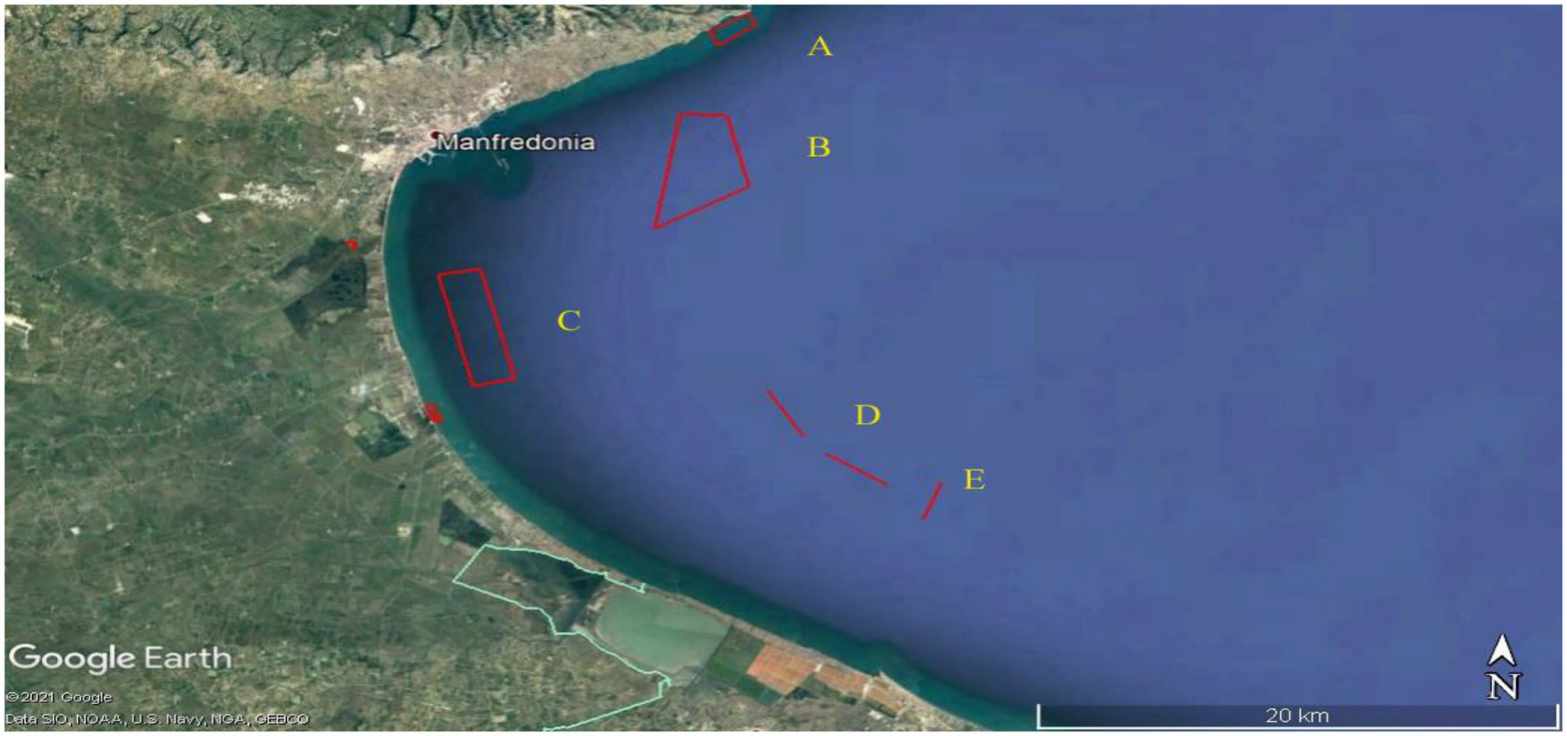
Five specific sampling areas (A, B, C, D, and E) of *Aphia Minuta* located along the Gulf of Manfredonia. Map Data: Google Earth ©2021/ Data SIO, NOAA, U.S. Navy, NGA, GEBCO.

*Aphia minuta* was collected in five specific areas (A, B, C, D, and E, Figure 1), located along the Gulf of Manfredonia, at different distances from the coast and with different natures of the seabed or surrounding biocenoses. The more abundant samples of *Aphia minuta* were collected in B, D, and E areas. **Area A** was chosen near the coast, 2.5 km from the southwestern limit of Mattinata Bay, and is characterized by dense meadows of Cymodocea nodosa on a sandy-muddy bottom, up to a depth of 7–9 meters. **Area B** is ~5 km from the coast, with depths between 11 and 13 m. The bottom is predominantly inconsistent but characterized by the presence of mud and bio-constructions. **Area C**, about 6 km away from the port of Manfredonia and 3 km at the closest point to the coast, has a depth of 11–13 m, a muddy bottom, and small formations of scattered bio-constructions. **Area D** is ~4.4 miles away, transverse from the coast, and ~14.3 miles from the port of Manfredonia, with depths between 18 and 20 m. It is featured by coral formations of limited development and detrital beds; the samplings were carried out only along the mud/sandy channels present among the environments mentioned. **Area E** concerns the Maërl and Rodoliti funds, overlooking the Saline di Margherita di Savoia.

The Gulf of Manfredonia is located along the Italian coast of the Adriatic Sea in the Apulia region. Many factors contribute to determining the oceanographic characteristics of the Gulf of Manfredonia, such as the WAC (Western Adriatic Current), which is powered by northeast winds and moves the water masses along the Italian coasts in a north vs. south direction. The climate of the area is temperate Mediterranean with alternating dry and rainy seasons. In the last 10 years, rainfall has not exceeded 700 mm/year, which determines the accumulation of nutrients in the river basin. In the event of torrential rains, large quantities of nutrient-rich water are discharged into the gulf, which can cause local eutrophication. Due to its physiographic characteristics, the Gulf of Manfredonia is characterized by shallow, low hydrodynamic, and very productive waters. The abundance of phytoplankton in the area is the basis of the high fish density.

According to the fishing season of the South Adriatic Sea and the current guidelines in Italy (Council Regulation No 1967/2006), samples were collected at each site at six different times of the year (*n* = 30): two sampling in winter (in January and February), two in spring (at March and April), and two sampling in Summer (at June and July). During each sampling, local fishing vessels from 10 to 40 m depth on the seabed follow the seasonal migrations and shoaling of *Aphia minuta*. A small meshed semipelagic trawl with three concentric bags coded from 16 to 5 mm (stretched) mesh size was used for the sampling. The chemical-physical parameters of the seawater were also recorded for each catch. At every sampling point, the samples of *Aphia minuta* were placed in self-draining polystyrene boxes, packed in flake ice, and delivered to the laboratory on the same day.

### 2.2 Nutritional analyses

#### 2.2.1 Chemical composition and fatty acid methyl esters profile

Fish samples were homogenized using a laboratory blender and frozen at −20°C until the subsequent analysis. The grounded mass was used as the representative sample for the analyses, and the results were expressed as a particular content/edible portion (EP).

Moisture, protein, lipid, and ash contents were performed according to AOAC methods (15), and all the chemical determinations were performed in triplicate.

The analysis for the determination of fatty acid composition was performed according to the method of O'Fallon et al. (16) with some modifications as previously described by Marino et al. (17). Briefly, 1 g of each sample was placed into a screw cap Pyrex reaction tube, added with 5.3 mL of MeOH, 0.7 mL of 10 N KOH in water, and 0.5 mg of C13:0/mL of the internal standard. Then, the tubes were incubated in a water bath at 55°C for 90 min, with handshaking for 5 s every 20 min. After incubation, the tubes were cooled to room temperature, and subsequently, 580 μL of 24 N H_2_SO_4_ were added. After cooling, 3 mL of hexane was added into each tube, vortexed, and then centrifuged at 500 × g (Eppendorf 5810 R, Hamburg, Germany) for 5 min at 21°C. The hexane layer containing the fatty acids methyl esters (FAME) was collected and transferred into a gas-chromatographic vial. The fatty acid profile was quantified through an Agilent 6890 N instrument (Agilent Technologies, Santa Clara, CA, USA) equipped with an HP-88 fused-silica capillary column (length 100 m, internal diameter 0.25 mm, film thickness 0.25 μm). Operating conditions were as follows: carrier gas (helium) at a constant flow of 1 mL/min; split-splitless injector at 260°C; split ratio 1:25; injected sample volume 1 μL; FID detector at 260°C. The temperature program of the column was 5 min at 100°C, then increased to 240°C (3.5°C/min) and held for 15 min. The retention time and area of each peak were computed using the 6890 N NETWORK GC system software. Fatty acids were identified by comparing their retention times with the fatty acid methyl standards (FIM-FAME-7-Mix, Matreya LLC, Pleasant Gap PA, USA), added of C18:1-11t, C18:2-9c,11t, C18:2-9c, 11c, C18:2-9t, 11t, and C18:2-10t, 12c (Matreya LLC, Pleasant Gap PA, USA). The fatty acid concentration was expressed as g fatty acids/100 g total fatty acids. Nutritional indices such as PUFA/SFA, n6/n3, % EPA+DHA, atherogenic and thrombogenic indices, and fish lipid quality were calculated according to Marino et al. (18).

All the chemical and fatty acid determinations were performed in triplicate.

#### 2.2.2 Amino acid determination

Amino acid extraction was carried out according to Marino et al. (19). Briefly, 20 mg of freeze-dried samples were placed in hydrolysis tubes with 500 μL of 6 M HCl. Tubes were sealed under vacuum and placed in a ventilated oven at 160°C for 75 min. Hydrolyzed samples were filtered through Whatman 0.45 μm filters, and filtered solutions were diluted 1:10 with ultrapure water before being submitted to automated online derivatization and injection. The HPLC system consisted of an Agilent 1260 Infinity Series chromatograph (Agilent Technologies, Santa Clara, CA, USA) equipped with a binary pump (G1312B), a diode-array detector (1315C), and a fluorescence detector (G1321B). The analyses were performed using a Zorbax Eclipse AAA column (150 × 4.6 mm i.d., 3.5 μm particles; Agilent Technologies, Palo Alto, CA, USA). Individual amino acid peaks were identified by comparing their retention times with those of standards. Results for amino acids were expressed as mg/100 g meat. Amino acid determinations were performed in triplicate.

### 2.3 Microbiological analyses

#### 2.3.1 Determination of antimicrobial activity of essential oils *in vitro*

Thymol (Sigma, Pool, UK), citrus extract (Biocitro, Probena s.l., Zaragoza, Spain), and lemon extract (Spencer Food Industrial, Amsterdam, The Netherlands) were tested to evaluate their antimicrobial activity against three specific spoilage microorganisms. Stock solutions (200–300–400–500 mg/L) of thymol were prepared in water-ethanol (1:1), while the other compounds were dissolved in distilled water. All solutions were freshly prepared before each use and sterilized by filtering through membranes (0.20 μm; Sartorius, Goettingen, Germany). The strains used were *Pseudomonas fluorescens* DSM 50090 purchased from a Public Collection (Deutsche Sammlung von Mikroorganismen und Zellkulturen), *Staphylococcus* spp., and *Escherichia coli* belonging to the culture collection of the Laboratory of Predictive Microbiology, University of Foggia. The microorganisms were stored at −20°C in nutrient broth (Oxoid, Milan, Italy) added with 33% sterile glycerol and grown under aerobic conditions in nutrient broth at 25°C for 48 h for *P. fluorescens* and at 37°C for 24 h for both strains. The effectiveness of natural compounds against fish spoilage microorganisms was determined by inhibition zone assays inoculating the substrates with 1 ml of cell suspension (10^6^ CFU/mL). For each natural compound, 0.1 mL of the active solutions, for each concentration, was poured into wells (9 mm diameter) previously cut with a sterilized cork-borer into the agar medium. The plates inoculated with *P. fluorescens* were incubated at 25°C for 48 h, while those with *Staphylococcus* spp. and *E. coli* were incubated at 37°C for 24 h. As a control for the antimicrobial activity of thymol, 0.1 ml of a water-ethanol (1:1) solution was poured into wells; for the other active compounds, 0.1 ml of distilled water was used. After incubation, the inhibition diameter was measured in three directions, and the average was tabulated. Three replications of this experiment were made.

#### 2.3.2 Challenge test in model system

Agar diskettes containing technical agar No. 3 (Oxoid, Milan) and 2% meat extract (Beef Extract, Oxoid) were used to simulate a fish filet model system. Before use, *P. fluorescens* and *E. coli* strains were grown in nutrient broth and incubated at 25°C for 48 h and 37°C for 24 h, respectively. The microorganisms were centrifuged at 4,000 x *g* for 10 min, the supernatant was discarded, and the pellet was suspended in a sterile saline solution (0.9% NaCl). After two dilutions of the stock solution, 200 μL of cell suspension was added to a test tube containing liquid agar and meat extract (19 mL; 55°C) and 1 mL of a 2 g/L citrus extract. Immediately after inoculation, the agar solution was put in Petri dishes and left to dry for 1 h; the concentration of citrus extract in the final sample was 100 mg/L, while the microorganisms were 3 log CFU/g. Samples were stored at 37°C for *E. coli* and 25°C for *P. fluorescens* and analyzed after 24, 48, and 72 h. Agar pieces (20 g) were taken and diluted in 180 mL of saline solution in a Stomacher bag (Seward, London, England), then homogenized for 1 min in a Seward Stomacher Lab Blender 400. Serial dilutions of the homogenates were inoculated on nutrient agar and incubated at 37°C for 24 h (*E. coli*) or 25°C for 48–72 h (*P. fluorescens*). All tests were performed in duplicate over two different batches. Samples not containing the citrus extract but inoculated with the test microorganisms were used as a control.

#### 2.3.3 Influence of citrus extract and different packages on the microbial stability of *Aphia minuta*

*Aphia minuta* samples were dipped for 5 min in a 100 mg/L citrus extract solution; after treatment, excess liquid was removed by air drying under a laminar flow hood. Samples immersed in distilled water were used as controls. Then, the samples were packaged in three different kinds of packaging materials: Nylon/Polyethylene bags (95 μm, Tecnovac, San Paolo D'Argon, Bergamo, Italy) using S100-Tecnovac equipment were 170 mm × 250 mm long, with O_2_ permeability of 50.65 cm^3^/m^2^ day atm and a water vapor transmission rate of 1.64 g/m^2^ day, as specified by the manufacturer; PLA polylactic bags [OTR: 45^*^10^3^ cc/(m^2^*day)]; corn starch based [OTR: 45^*^10^3^ cc/(m^2^*day)] under three conditions: (A) under vacuum (UV); (B) ordinary Atmosphere (AO); and (C) modified atmosphere (65% N_2_-30% CO_2_-5% O_2_; MA). Samples were stored at 4°C for 7 days and periodically analyzed as described in the following. All analyses were conducted twice.

For microbiological analyses, the following media were used: plate count agar (PCA) incubated at 5°C for 1 week and at 30°C for 48 h under aerobic conditions for psychrophilic microorganisms and total bacteria count, respectively; Baird Parker Agar Base, supplemented with Egg Yolk supplement, for coagulase-positive staphylococci; Pseudomonas agar base (PAB) supplemented with Pseudomonas CFC supplement, incubated at 25°C for 48 h for Pseudomonadaceae; Violet Red Bile Glucose Agar (VRBGA) incubated at 37°C for 24 h for Enterobacteriaceae. All the media and the supplements were sourced from Oxoid (Milan, Italy).

### 2.4 Statistic

Nutritional properties data were subjected to analysis of variance (ANOVA) using the GLM procedure of the SAS statistical software (20). The mathematical model included the fixed effect of sampling season and random residual error. All effects were tested for statistical significance (to *P* < 0.05), and significant effects were reported in tables. When significant differences were found (at *P* < 0.05 unless otherwise noted), Tukey's test was performed for multiple comparisons among means.

For microbiological studies, significant differences were pointed out through a *t*-test for paired comparison, while ANOVA was used for the challenge test, using the time, the packaging and modified atmosphere, and treatment (control vs. dipping in citrus extract) as categorical predictors.

## 3 Results and discussion

### 3.1 Chemical composition and fatty acid profile

The chemical composition of *Aphia minuta* as affected by sampling season is shown in Table 1. No significant differences were found for fat and ash contents during the seasons, remaining relatively stable throughout the year (0.98–1.20 and 3.19–3.66% for fat and ash, respectively). A significant seasonal variation concerning protein (14.56–16.09%) and moisture content (78.85–80.91%) was noticed. Higher protein content was found in spring (*P* < 0.01) compared to the other seasons, while moisture content in this season showed the lowest content (*P* < 0.01). The highest value of protein observed in spring coincides with the high feeding period of *Aphia minuta*, as there is a peak in the primary production (planktonic blooms) during spring and summer. Particularly, *Aphia minuta* has a diet consisting of zooplankton (decapods, crustaceans, copepods, and cirripedes), a protein source.

**Table 1:** Chemical composition (%) of *Aphia minuta* as affected by the sampling season (means ± SEM).

	**Sampling season**				
	**Winter (*****n*** = **10)**	**Spring (*****n*** = **10)**	**Summer (*****n*** = **10)**	**SEM**	**Effect**, ***P***
Moisture	79.70 ab	78.85 b	80.91 a	0.45	^**^
Fat	1.20	1.14	0.98	0.12	NS
Protein	15.86 ab	16.09 a	14.66 b	0.32	^**^
Ash	3.09	3.66	3.10	0.18	NS

NS, not significant.

^**^P < 0.01.

Letters indicate significant differences in a row.

The composition of *Aphia minuta's* fatty acids during the winter, spring, and summer seasons is shown in Table 2. The percentage of saturated fatty acids (SFA) did not change during the winter, spring, and summer seasons, while monounsaturated and polyunsaturated fatty acid content was affected by sampling season (*P* < 0.05 and *P* < 0.01, respectively). Monounsaturated fatty acids (MUFA) were higher in winter compared to the percentage found in spring and summer seasons, with the highest content of oleic acid (*P* < 0.01). The content of polyunsaturated fatty acids (PUFA) was the highest in spring and summer. Particularly during summer, *Aphia minuta* showed the lowest content of n-6 PUFA and the highest content of n-3 PUFA. In contrast, the highest content of n-6 and the lowest content of n-3 PUFA were found in the winter. Among n-3 PUFAs, linolenic, eicosapentaenoic (EPA; C20:5 n-3), docosapentaenoic (DPA; C22:5 n-3), and docosaeasoenoic (DHA; C22:5 n-3) fatty acids showed a higher value (*P* < 0.01) in spring and summer compared to the winter season.

**Table 2:** Fatty acid profile (%) of *Aphia minuta* as affected by the sampling season (means ± SEM).

	**Sampling season**				
	**Winter (*****n*** = **10)**	**Spring (*****n*** = **10)**	**Summer (*****n*** = **10)**	**SEM**	**Effect**, ***P***
C12:0	0.05	0.05	0.11	0.05	NS
C14:0	3.31	2.98	3.53	0.19	NS
C15:0	0.63	0.55	0.54	0.10	NS
C16:0	14.80	14.84	14.27	0.28	NS
C17:0	1.43	1.38	1.55	0.12	NS
C18:0	4.15	3.96	3.95	0.15	NS
C21:0	0.34	0.25	0.31	0.06	NS
Other SFA	0.49	0.36	0.49	0.05	NS
SFA	25.20	24.37	24.75	0.45	NS
C16:1	3.80	3.34	3.75	0.18	NS
C17:1	0.11	0.13	0.10	0.03	NS
C18:1t9	0.16	0.11	0.15	0.03	NS
C18:1c9	5.32 a	4.55 b	3.95 b	0.31	^*^
C20:1	0.26	0.25	0.30	0.05	NS
Other MUFA	0.42	0.19	0.18	0.09	NS
MUFA	10.08 a	8.57 b	8.43 b	0.41	^*^
C18:2t9t12	0.07	0.05	0.05	0.03	NS
C18:2c9c12	0.94	0.89	0.91	0.11	NS
C20:2n6	5.58 a	5.24 ab	4.75 b	0.25	^**^
C20:3n6	0.01	0.03	0.02	0.01	NS
C20:4n6	0.64 a	0.50 b	0.48 b	0.02	^**^
C22:2n6	0.04	0.02	0.05	0.02	NS
C18:3n3	0.84 b	1.28 a	1.32 a	0.15	^**^
C20:3n3	0.05 b	0.13 a	0.16 a	0.02	^*^
C20:5n3 (EPA)	16.80 b	17.63 a	17.75 a	0.23	^**^
C22:6n3 (DHA)	39.74 b	41.27 a	41.32 a	0.25	^***^
n6	7.28 a	6.74 ab	6.26 b	0.19	^**^
n3	57.43 b	60.31 a	60.55 a	0.28	^**^
PUFA	64.71 a	67.05 b	66.81 b	0.31	^**^

NS, not significant.

^*^P < 0.05.

^**^P < 0.01.

^***^P < 0.001.

Other SFA = C 20:0, C22:0, C23:0, C24:0; other MUFA = C 14:1, C15:1, C22:1, C24:1.

Letters indicate significant differences in a row.

As reported in a previous study, the fatty acid composition of fish varies throughout the year due to factors such as the fish's life cycle and external influences, such as their food's temperature, salinity, and fatty acid composition (21). The seasonal changes in the fatty acid profile observed in *Aphia minuta* are consistent with variations observed in European sardine (*Sardina pilchardus*), which show the highest levels of PUFA and n-3 PUFA during intensive feeding in the summer season (22). In particular, European sardine is recognized as a highly nutritious and important species of Mediterranean Sea fisheries (23). It is popular for its rich of long-chain polyunsaturated fatty acids. The n-3 PUFA levels found in *Aphia minuta* are higher than those reported in sardines.

Regardless of the sampling season, it is worth noting that in *Aphia minuta*, the content of palmitic acid (C16:0) was lower than other little fishes of the Adriatic Sea. In particular, previous studies reported palmitic acid as the most abundant fatty acid in sardine in anchovy and picarel (24). On the contrary, in the *Aphia minuta*, the fatty acids with the highest percentages were DHA and EPA, resulting in 88% of the total polyunsaturated fatty acids. This is an important result from a nutritional point of view because n-3 eicosapentaenoic (EPA) and docosahexaenoic (DHA) fatty acids are essential for human development and have different beneficial effects on human health, performing important anti-inflammatory and antithrombotic functions and playing a very important role in the prevention and treatment of coronary heart disease (25). In contrast, palmitic acids are positively associated with a risk of atherosclerosis and other cardiovascular diseases.

The positive effects of n-3 PUFA on human health are related to the actual dietary intake of n-3 PUFA. In the present study, 100 g of *Aphia minuta* provided nearly 1,800 mg of EPA+ DHA. On a quantitative basis, the values of this fish accounted for 90% of the recommended intake of long-chain (LC) n-3 PUFA [2g EPA+ DHA per day; (26)]. Thus, *Aphia minuta* can be considered a high source of n-3 and a very good choice of fish all year round.

Based on the fatty acid profile, nutritional indices were calculated (Table 3). Samples of *Aphia minuta* collected in spring and in summer showed higher values of P/S (*P* < 0.05), EPA+DHA percentage (*P* < 0.01), and fish lipid quality index (*P* < 0.05) than samples collected during the winter season, while n-6/n-3 was the lowest in summer sampling (*P* < 0.05). This result confirms that *Aphia minuta* fished in spring and summer had better nutritional properties. It has been demonstrated that FAs play positive or negative roles in the prevention and treatment of diseases; in the last years, many studies (18, 27) have been addressed to evaluate the nutritional value of fatty acids and to explore their potential usage in disease prevention and treatment to accurately select appropriate indices.

**Table 3:** Nutritional indices of *Aphia minuta* as effected by the sampling season (means ± SEM).

	**Sampling season**				
	**Winter (*****n*** = **10)**	**Spring (*****n*** = **10)**	**Summer (*****n*** = **10)**	**SEM**	***P*** **, effect**
P/S	2.57 b	2.75 a	2.70 a	0.05	^*^
n6/n3	0.13 a	0.11 ab	0.10 b	0.01	^*^
Atherogenic index (AI)	0.38	0.35	0.38	0.04	NS
Thrombogenic index (TI)	0.12	0.11	0.11	0.04	NS
EPA+DHA (%)	56.54 b	58.90 a	59.07 a	0.18	^**^
Fish lipid quality (FQL)	224.38 b	241.74 a	238.67 a	1.55	^*^

NS, not significant.

^*^P < 0.05.

^**^P < 0.01.

^***^P < 0.001.

AI = (C12:0 + 4 × C14:0 + C16:0)/(MUFA + Σn6 + Σn3).

TI = (C14:0 + C16:0 + C18:0)/[(0.5 × MUFA) + (0.5 × Σn-6 PUFA) + (3 × Σn-3 PUFA) + (Σn-3 PUFA/Σn-6 PUFA)].

FLQ = 100 × (C20:5n-3 + C22:6n-3)/(Σ Saturated Fatty Acids).

Letters indicate significant differences in a row.

The n-6/n-3 ratio is an important index for determining the quality of fat, as a higher amount of n-6 fatty acids promotes the pathogenesis of many diseases, including cancer, inflammatory, and autoimmune diseases, whereas increased levels of n-3 PUFA exert suppressive effects (28). Reducing the ratio of n-6/n-3 fatty acids in the human diet was essential to help prevent coronary heart disease and reduce the risk of cancer (28). In the current study, the ratio of n-6/n-3 fatty acids found in Aphia minuta ranged from 0.10 to 0.13; this value is much lower compared to previous studies (29) and to the threshold value (< 4) indicated by the World Health Organization (WHO). Similarly, Atherogenic and Thrombogenic indices ranged from 0.35 to 0.38 and from 0.11 to 0.12, respectively, showing values much lower than the threshold values (atherogenic index < 0.5 and thrombogenic index < 1.0) suggested as desirable to minimize the risks of cardiovascular diseases.

### 3.2 Amino acids

Next to providing essential fatty acids, fish is a source of high-quality proteins; particularly, fish protein is considered a complete source of protein, as it is only the protein source that contains a well-balanced amino acid composition that has eight essential and eight non-essential amino acids (30).

The amino acid composition of *Aphia minuta* during the winter, spring, and summer seasons is shown in Table 4. The highest content of essential amino acids was measured during the spring and summer (*P* < 0.01), with leucine and lysine being the most dominant amino acids. Essential amino acids are basic in the diet, particularly for certain populations with specific needs, such as those of children and sick and old people. In particular, leucine can stimulate the synthesis of muscle protein, while lysine has been shown to improve growth performance in the body (31).

**Table 4:** Aminoacids composition (mg/100 g fish) of *Aphia minuta* as affected by the sampling season (means ± SEM).

	**Sampling season**				
	**Winter (*****n*** = **10)**	**Spring (*****n*** = **10)**	**Summer (*****n*** = **10)**	**SEM**	**Effect**, ***P***
Arginine	969.6	977.7	936.6	22.3	NS
Histidine	452.4	395.1	382.0	24.7	NS
Threonine	924.2	995.4	949.2	44.7	NS
Valine	876.7	946.6	906.9	38.9	NS
Methionine	713.3	746.9	744.2	22.6	NS
Phenylaalnine	629.2	688.7	696.6	31.3	NS
Isoleucine	740.5	839.0	849.7	42.8	NS
Leucine	1,622.2 B	1,852.7 a	1,938.3 a	66.5	^*^
Lysine	1,865.5 B	2,079.0 a	2,159.0 a	77.5	^**^
Aspartate	1,551.0	1,618.5	1,634.5	63.5	NS
Glutamate	2,112.4	2,170.6	2,275.6	76.5	NS
Serine	571.9	536.5	504.6	55.6	NS
Glycine	722.7	682.2	641.5	45.8	NS
Alananine	908.8	990.4	1,100.5	48.7	NS
Tyrosine	618.7	553.4	536.3	31.5	NS
Cystein	336.5	247.3	263.0	25.5	NS
Proline	391.3	480.5	404.4	22.5	NS
AAT	15,056.8 B	16,104.4	15,242.8 b	250.5	^*^
EAAT	8,793.4 B	9,521.2 a	9,562.5 a	99.5	^**^
NEAAT	7,213.4	7,279.2	7,360.3	68.8	NS
EAA/NEAA	1.2	1.3	1.3	0.3	NS
**%EA/AAT**	58.4 B	59.1 b	62.7 a	0.65	^*^

Letters indicate significant differences in a row.

NS, not significant.

^*^P < 0.05.

^**^P < 0.01.

^***^P < 0.001.

AAT, amino acid total; EAAT, essential amino acid total; NEAAT, non-essential amino acid total.

The highest value was found in spring (*P* < 0.05) related to the total amino acid content. This result is consistent with the highest protein percentage found in this season. It is worth noting that *Aphia minuta* showed a very high percentage of essential amino acids/total amino acids, ranging from 58.4 to 62.7%, with the highest ratio in summer (*P* < 0.05). Furthermore, *Aphia minuta* has a high arginine content; being a functional amino acid, it plays an important role in the regulation of metabolic pathways to improve health, survival, growth, development, lactation, and reproduction of the organisms (32). These results highlight that the amino acid content of the *Aphia minuta* has many similarities with the amino acid profile of sardines (22), which is a rich source of essential and functional amino acids.

### 3.3 Microbiology

The antimicrobial activity of the three natural compounds at different concentrations (200–500 mg/L) was measured against three well-defined fish spoilage/pathogen bacteria, used as targets for the following reasons: *P. fluorescens*, such ad all pseudomonads, is among the most resistant strains to EOS, while *E. coli* and *Staphylococcus* spp. are hygiene indicators. The diameter of the growth inhibition zone (measured in mm) was used as the criterion for antimicrobial activity. Table 5 shows the inhibitory effect of the active compounds on the growth of the target strains. The inhibitory effect of thymol was not observed against *Staphylococcus* spp., whereas for citrus and lemon extracts, the zone of inhibition was between 2 and 2.5 mm at all concentrations tested. For *P. fluorescens* and *E. coli*, at the lowest concentrations (200–300 mg/L) of citrus and lemon extracts, the inhibition zone was around 1.5–2 mm, without statistically significant differences between the compounds used. On the other hand, thymol concentrations up to 500 mg/L were required to obtain the same antibacterial activity.

**Table 5:** Inhibition effects of natural compounds tested against *Pseudomonas fluorescens, Staphylococcus* spp., and *Escherichia coli*.

**Natural compound**	**Target strain**	**Diameter of the zones of inhibition (mm)**			
		**200 mg/L**	**300 mg/L**	**400 mg/L**	**500 mg/L**
Thymol	*Pseudomonas fluorescens*	0.87 ± 0.12	0.93 ± 0.06	1.17 ± 0.06	1.40 ± 0.17
	*Staphylococcus* spp.	-	-	-	-
	*Escherichia coli*	0.63 ± 0.06	0.73 ± 0.06	1.10 ± 0.20	1.13 ± 0.15
Citrus extract	*Pseudomonas fluorescens*	1.30 ± 0.40	1.60 ± 0.12	2.00 ± 0.00	2.20 ± 0.20
	*Staphylococcus* spp.	2.30 ± 0.17	2.20 ± 0.17	2.20 ± 0.20	2.13 ± 0.15
	*Escherichia coli*	2.90 ± 0.10	1.80 ± 0.12	2.00 ± 0.00	2.03 ± 0.06
Lemon extract	*Pseudomonas fluorescens*	1.60 ± 0.00	1.77 ± 0.25	1.93 ± 0.12	2.13 ± 0.12
	*Staphylococcus* spp.	2.50 ± 0.00	2.43 ± 0.12	2.23 ± 0.25	2.67 ± 0.29
	*Escherichia coli*	2.00 ± 0.00	2.00 ± 0.00	2.07 ± 0.12	2.00 ± 0.00

The mean values ± standard deviation.

The antibacterial activity of citrus and lemon extract was concentration-independent, as their antibacterial activity did not increase proportionally with increasing concentrations. In fact, against *Staphylococcus* spp. and *E. coli*, the inhibitory effect of these natural compounds was independent of the concentration, and the diameter of the inhibition zone was ~2–2.5 mm by using 200, 300, 400, or 500 mg/L. The trend of thymol was different from the other active compounds; the inhibition zone was very low, up to 300 mg/L, while at the highest concentration, an inhibition diameter of about 1.5 mm was recorded. Thymol is one of the most known and widely studied EO, extracted among others from *Thymus vulgaris* and *Ocimum gratissium* and with a variety of biological functions (antimicrobial, anti-inflammatory, antioxidant, antimutagenic, larvicidal, analgesic, and radioprotective effects) (33). Although many authors in the past suggested a higher sensitivity of Gram-negative microorganisms (34, 35), the results hereby collected confirm the findings of the meta-analysis of Speranza et al. (36), who found a strong resistance of some Gram-negative bacteria, mainly those belonging to *E. coli* species and *Pseudomonas* genus.

On the other hand, the antimicrobial activity recorded by citrus and lemon extracts confirms the potentiality of oils and active components extracted from *Citrus* spp.; some authors reported in the past the ability to eradicate several *Pseudomonas* and/or *E. coli* strains by different mechanisms, including among others the disturbance of quorum sensing, biofilm eradication, or affecting cell surface hydrophobicity (37–39).

**Figure 2:**
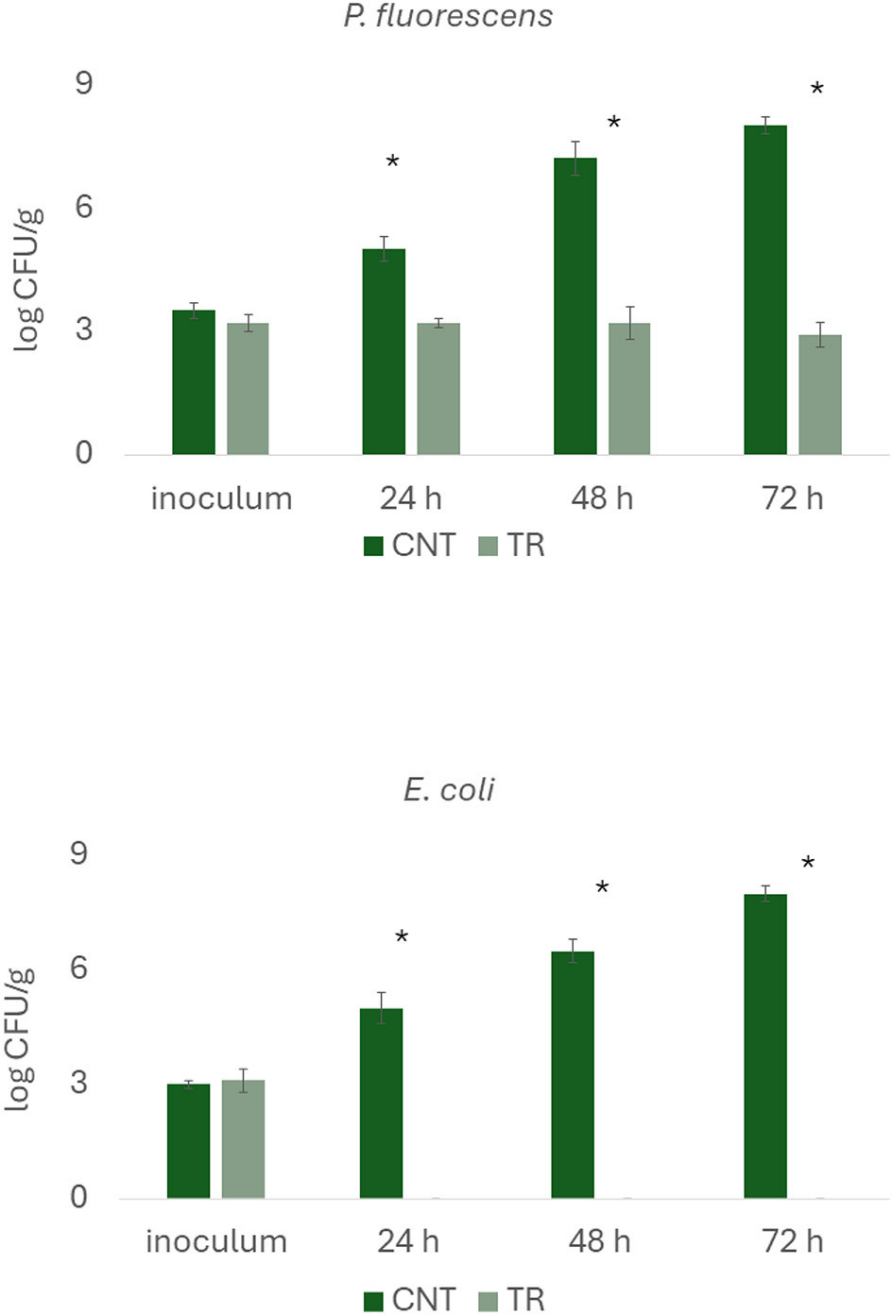
The concentration of *Pseudomonas fluorescens* and *Escherichia coli* in model systems (mean ± standard deviation). CNT, control; TR, sample dipping in citrus extract solution. *This symbol denotes a statistical difference between control and treated samples (*t*-test, *P* < 0.05).

Based on these findings, lemon and citrus extracts were regarded as promising antimicrobials for the second step, but lemon extract was not used in the second phase of the experiment because its antimicrobial activity was not reproducible due to its variable and complex composition; in fact, some preliminary analyses by authors revealed that its antimicrobial activity changed as a function of the batch used. Furthermore, the organoleptic impact on the matrix should not be underestimated when using essential oils. Therefore, based on the preliminary results, the citrus extract was chosen for the next steps, as the commercial preparation used is less organoleptically harmful, as also found by authors for other applications. Thus, a solution of citrus extract at 200 mg/L was chosen to study the antimicrobial efficacy on the development kinetics of the two target microorganisms. The test was conducted in a model system at this stage on two representative microorganisms of spoiling (*P. fluorescens*) and pathogenic (*E. coli*) microbiota. In the citrus extract sample, the concentration of *P. fluorescens* was about 3 log CFU/g throughout time, while in the control, the concentration increased from 3.5 to 8.0 log CFU/g after 72 h. As for *E. coli*, the pathogen was already below the detection threshold after 24 h, while the cellular concentration of the microbial target in the control sample was 8.1 log CFU/g after 72 h (Figure 2). These results suggest a possible bacteriostatic effect of citrus extract against *Pseudomonas* spp. and a bactericidal effect against *E. coli*.

The difference between the two test strains, at least for the action on the growth/death kinetic, confirms the possibility that an EO could act differently, depending on various conditions, but mainly due to the characteristics of the external layers. In the case of *E. coli*, several authors postulated different modes of action. For example, Álvarez-Ordóñez et al. (40) found that a commercial citrus extract determined the formation of pores with the leakage of cellular components due to the interaction with the carboxylic groups of the fatty acids of the membrane. On the other hand, the same extract used in this study was tested by de Nova et al. (41), who also found a morphological alternation of the cell envelope. These changes, along with possible changes in the hydrophobicity of the cell surface (38), would have been responsible for the bactericidal effect on *E. coli*. In the case of *Pseudomonas* spp., different mechanisms have been postulated, but the most important ones are the reduction of cell motility and the disturbance of cell-to-cell communication, which could determine a delay rather than inactivation (42).

**Figure 3:**
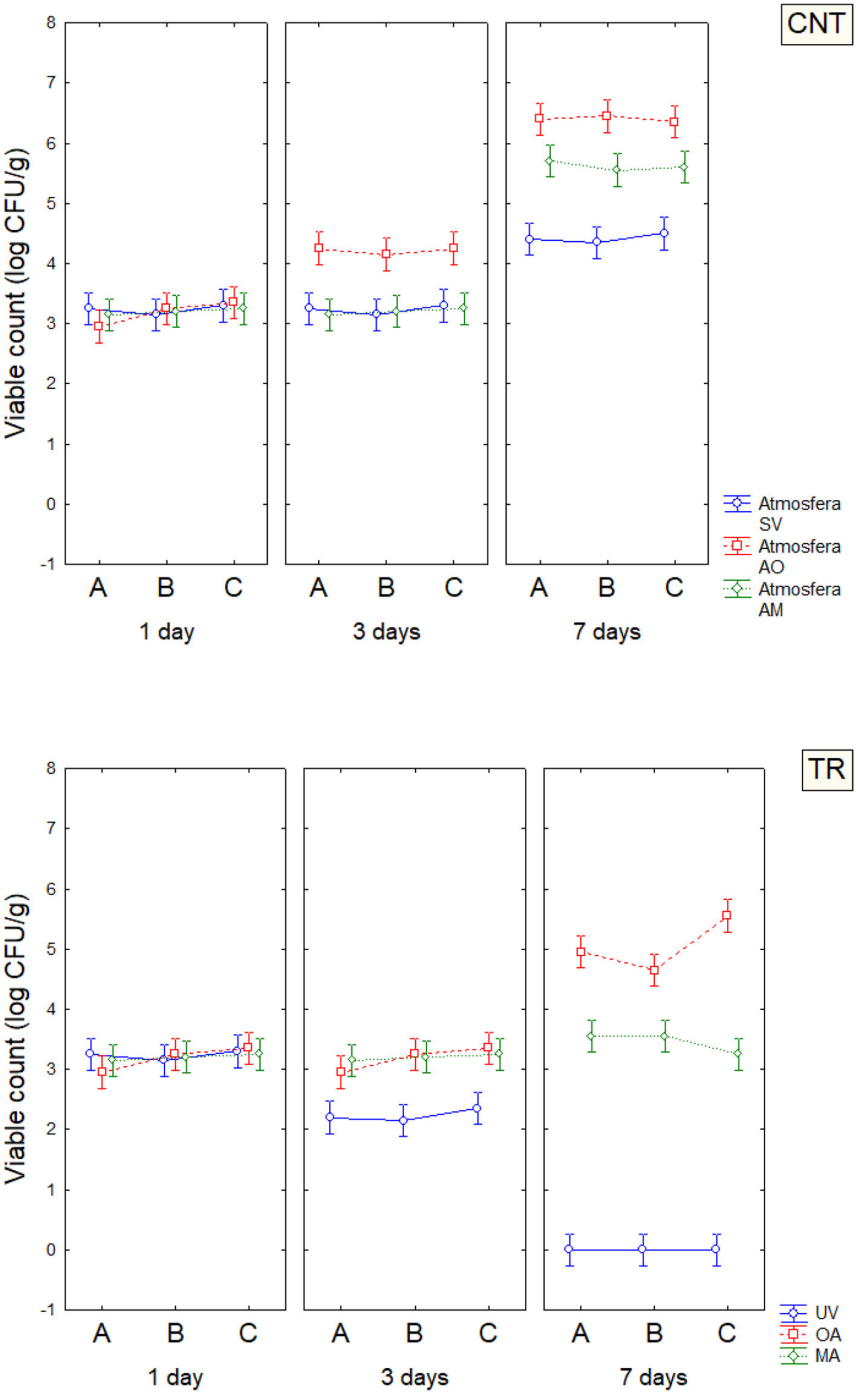
The concentration of psychrophilic microorganisms in *Aphia minuta* samples during the shelf-life test; error bars represent 95% confidence intervals. UV, vacuum; OA, ordinary atmosphere; MA, modified atmosphere (65% N_2_, 30% CO_2_, and 5% O_2_). CNT, control; TR, sample dipping in citrus extract solution. A, conventional bags; B, PLA bags; C, corn starch bags.

**Figure 4:**
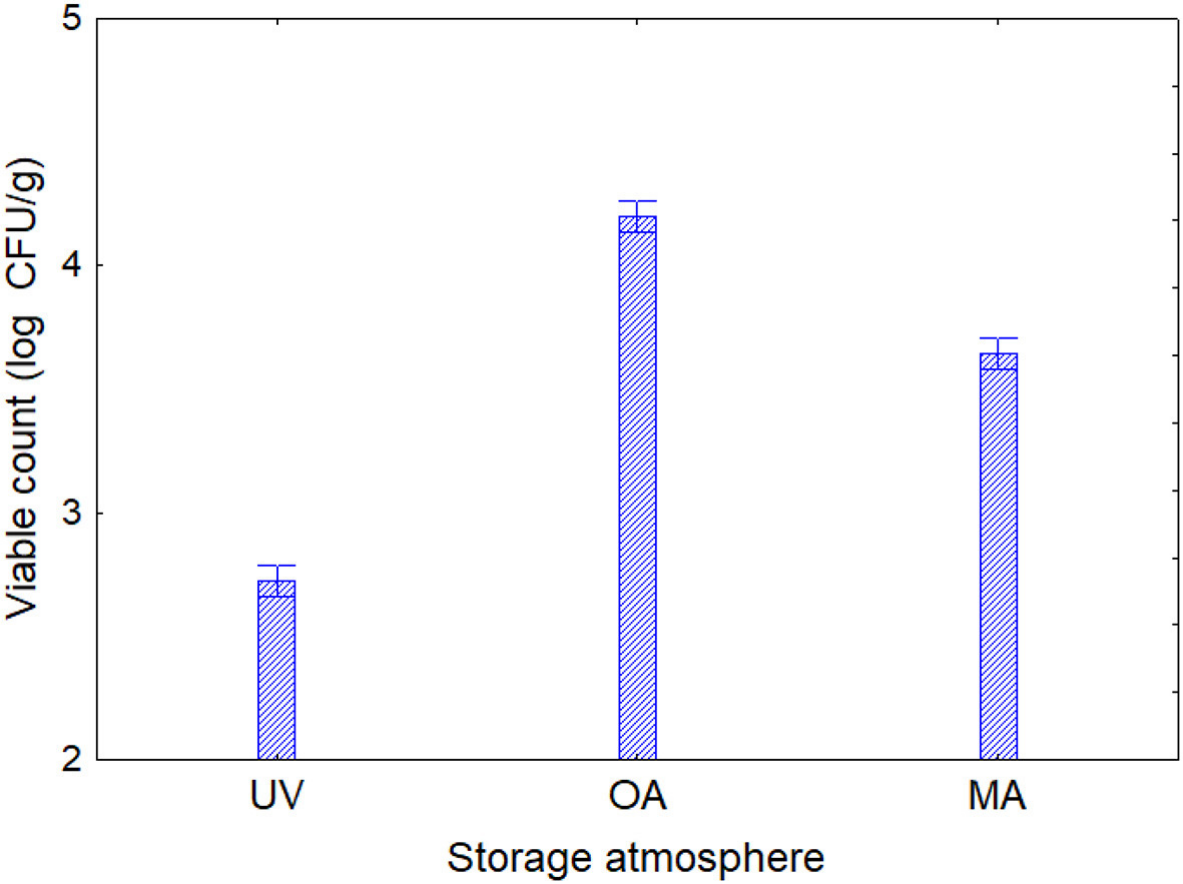
Decomposition of the statistical hypothesis for the effect of the storage atmosphere on the concentration of psychrophilic microorganisms during the shelf-life test. Error bars represent the 95% confidence interval. UV, vacuum; OA, ordinary atmosphere; MA, modified atmosphere (65% N_2_, 30% CO_2_, and 5% O_2_).

**Figure 5:**
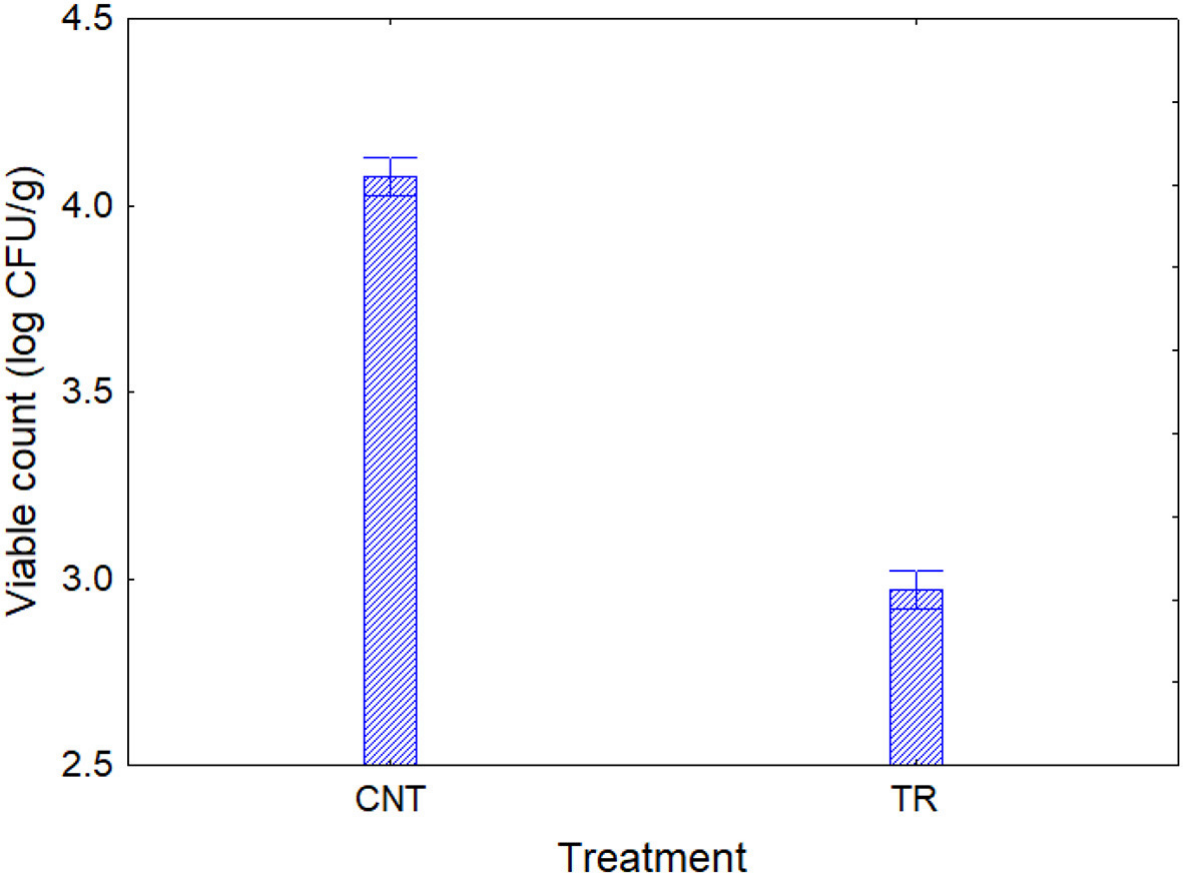
Decomposition of the statistical hypothesis for the treatment on the concentration of psychrophilic microorganisms during the shelf-life test. Error bars represent the 95% confidence interval. CNT, control sample; TR, sample dipping in the citrus extract solution.

Based on the results obtained in the model system, a shelf-life test was carried out. Enterobacteriaceae, staphylococci, and SSO contamination were random and variable, neither affected by treatment conditions nor packaging, as many samples were below the detection limit. On the other hand, psychrophilic microorganisms showed consistent developmental kinetics that were responsive to the input variables and allowed accurate assessment of the microbiological quality of the product; this result is of concern, as it is well-known that psychrophilic microorganisms are limiting for the quality and the shelf life of fish (43–45). Data on the cellular concentration of psychrophilic microorganisms in *Aphia minuta* samples during the shelf-life test are shown in Figure 3. In the control samples, there was an increase in the concentration of psychrophilic microorganisms over time (4–6 log CFU/g), which was mainly dependent on the type of storage atmosphere, as after 7 days the samples UV-packed showed a significantly lower viable count (4.2–4.5 vs. 6.2–6.5 log CFU/g in AO samples); these results are probably because pseudomonads represent a significant proportion of psychrophilic microbiota in fish (46), and the removal of oxygen could exert a detrimental effect as they are aerobic. Under vacuum, the citrus extract exerted a biocidal effect, as the target microbes were below the detection threshold after 7 days, due probably to the combination of oxygen removal and the antimicrobial effect of EO. To better understand the effects of the variables, MANOVA modeled the data; the table of standardized effects shows that all predictors were significant as single terms but with different statistical weights (Table 6). The most significant predictor variable was the dipping treatment (*F*-test, 926.44), followed by the storage atmosphere (*F*-test, 569.86), storage time (*F*-test, 301.25), and packaging type (*F*-test, 4.39). In addition, statistical analysis showed the significance of some interactive terms (in order of statistical weight: dipping treatment^*^storage time, storage atmosphere^*^storage time, storage atmosphere^*^dipping treatment, storage atmosphere^*^dipping treatment^*^storage time). Estimating the quantitative effects of predictors is possible by decomposing the statistical hypothesis, which does not show real trends but the mathematical correlation of each predictor vs. the dependent variable. Figures 4, 5 show the decomposition of the statistical hypothesis for the individual effects of predictors (storage atmosphere, treatment). As expected, the most effective storage atmosphere was UV (2.7 log CFU/g), followed by MA (3.6 log CFU/g), and finally, AO (4.3 log CFU/g; Figure 4). Regarding the effect of the treatment (Figure 5), the cell concentration of psychrophilic microorganisms was 4.1 log CFU/g in the control and 3.1 log CFU/g in the treated sample, indicating a very strong quantitative effect.

**Table 6:** Standardized effects of packaging, dipping in citrus extract solution (treatment), storage time, and atmosphere.

**Variable**	**Fisher test**
{1} Packaging	4.39
{2} Storage atmosphere	560.86
{3} Treatment	926.44
{4} Storage time	301.25
Packaging-Storage atmosphere	-
Packaging-Treatment	-
Storage atmosphere-Treatment	92.38
Packaging-Storage time	-
Storage atmosphere-Storage time	295.91
Treatment-Storage time	475.10
Packaging-Storage atmosphere-Treatment	-
Packaging-Storage atmosphere-Storage time	-
Packaging-Treatment-Storage time	-
Storage atmosphere-Treatment-Storage time	74.14
1-2-3-4	-

## 4 Conclusion

This study provides valuable insights into the nutritional values, fatty acid and amino acid profiles, and the shelf life of *Aphia minuta* collected from the Adriatic Sea.

*Aphia minuta* is a rich source of n-3 fatty acids and amino acids. It has the highest content of n-3 fatty acids and essential amino acids during spring and summer. Additionally, it maintains a very high content of eicosapentaenoic and docosahexaenoic fatty acids throughout the year. These acids are essential for human development and offer various beneficial effects on human health.

Concerning the microbiological quality, psychrophilic microbiota could present an issue. However, combining natural antimicrobial compounds and packaging could be a valuable strategy to prolong the shelf life. Experiments conducted in the model system suggest that the effect of the citrus extract could be either bacteriostatic or bactericidal, depending on the target microorganisms. Nevertheless, this is a promising strategy. Specifically, the combination of UV packaging and dipping in a citrus extract solution at 100 mg/L inhibited the growth of psychrophilic microorganisms, reducing their viable count below the detection limit after 7 days. However, the packaging material did not exert a significant effect under the conditions used in this research.

In conclusion, the information and results reported in this study could be very useful for exploiting and expanding the market of the *Aphia minuta* and paving the way for new valorization strategies.

## Data availability statement

The data analyzed in this study is subject to the following licenses/restrictions. The datasets for this study are available upon request to interested researchers. Requests to access these datasets should be directed to RM, rosaria.marino@unifg.it.

## Ethics statement

The animal study was approved by Institutional Animal Care and Use Committee of University of Foggia (protocol number: 006-2022). The study was conducted in accordance with the local legislation and institutional requirements.

## Author contributions

RM: Conceptualization, Funding acquisition, Investigation, Methodology, Project administration, Writing – original draft, Writing – review & editing. MA: Supervision, Validation, Visualization, Writing – review & editing. AM: Formal analysis, Software, Writing – review & editing. AR: Formal analysis, Software, Writing – original draft. BS: Validation, Visualization, Writing – review & editing. AB: Conceptualization, Investigation, Methodology, Project administration, Writing – review & editing.
